# Nicotinamide riboside supplementation is not associated with altered methylation homeostasis in Parkinson’s disease

**DOI:** 10.1016/j.isci.2023.106278

**Published:** 2023-02-27

**Authors:** Johannes J. Gaare, Christian Dölle, Brage Brakedal, Kim Brügger, Kristoffer Haugarvoll, Gonzalo S. Nido, Charalampos Tzoulis

**Affiliations:** 1 Neuro-SysMed Center, Department of Neurology, Haukeland University Hospital, Bergen, Norway; 2 K.G Jebsen Center for Translational Research in Parkinson’s Disease, University of Bergen, Bergen, Norway; 3 Department of Clinical Medicine, University of Bergen, Bergen, Norway

**Keywords:** Biological sciences, Neuroscience, Molecular neuroscience, Clinical neuroscience

## Abstract

Replenishing nicotinamide adenine dinucleotide (NAD) via supplementation of nicotinamide riboside (NR) has been shown to confer neuroprotective effects in models of aging and neurodegenerative diseases, including Parkinson’s disease (PD). Although generally considered safe, concerns have been raised that NR supplementation could impact methylation dependent reactions, including DNA methylation, because of increased production and methylation dependent breakdown of nicotinamide (NAM). We investigated the effect of NR supplementation on DNA methylation in a double blinded, placebo-controlled trial of 29 human subjects with PD, in blood cells and muscle tissue. Our results show that NR had no impact on DNA methylation homeostasis, including individuals with common pathogenic mutations in the *MTHFR* gene known to affect one-carbon metabolism. Pathway and methylation variance analyses indicate that there might be minor regulatory responses to NR. We conclude that short-term therapy with high-dose NR for up to 30 days has no deleterious impact on methylation homeostasis.

## Highlights

• NR 1000 mg/day for 30 days does not alter the global DNA methylation profile in blood
• NR does not impact DNA methylation in individuals carrying common *MTHFR* mutations
• Pathway and methylation variance analyses indicate minor regulatory responses to NR

## Graphical abstract

**Figure:**
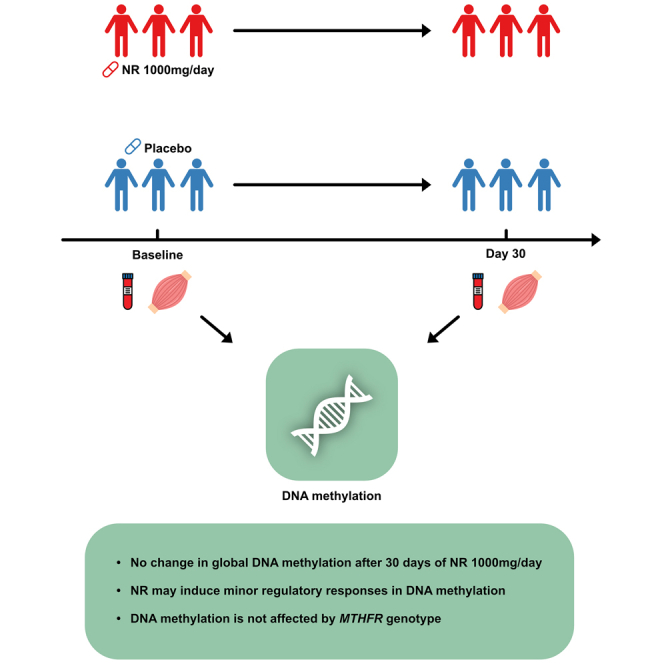

## Introduction

Nicotinamide adenine dinucleotide (NAD) is a coenzyme required for metabolic oxidoreduction reactions integral to cellular energy metabolism, including glycolysis, fatty acid β-oxidation, Krebs cycle and oxidative phosphorylation (OXPHOS). In addition, the oxidized form of NAD, NAD^+^, is a substrate for a number of vital non-redox reactions involved in, among others, DNA repair, histone- and other protein deacylation reactions, and second messenger generation.^1^^,^^2^ These reactions cause NAD^+^ degradation and thus constant NAD replenishment is important for cell survival. NAD levels decline with age and this has been proposed to contribute to age-related diseases, including Alzheimer's disease, Parkinson's disease (PD) and amyotrophic lateral sclerosis.^1^^,^^2^^,^^3^

Replenishing NAD levels via the supplementation of precursors, and/or increasing the NAD^+^/NADH ratio (e.g., via caloric restriction) have been shown to prolong lifespan and healthspan in animals, as well as to provide neuroprotection in models of neurodegenerative disorders.^2^^,^^4^^,^^5^ Encouraged by robust preclinical evidence, the therapeutic potential of drugs augmenting NAD-metabolism, most notably NAD precursors, is being increasingly investigated in clinical studies.^6^ In recent years, nicotinamide riboside (NR) has emerged as a safe^7^^,^^8^ and widely used NAD precursor, which is currently or has been applied in more than 70 registered clinical trials on a wide range of diseases, including lifestyle disorders and age-related neurodegenerative disorders.^9^ Moreover, we recently showed that oral NR supplementation augments NAD levels in the brain of individuals with PD, an effect that is associated with altered cerebral glucose metabolism and a mild clinical improvement after 30 days.^10^

**Figure 1:**
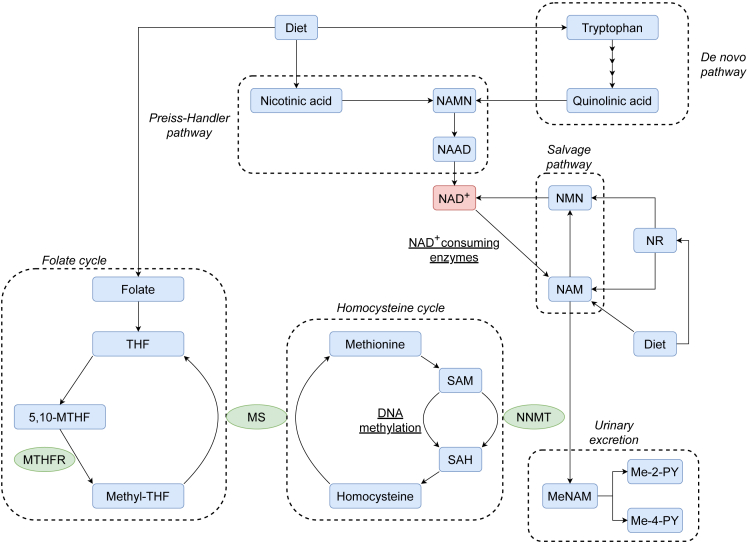
Simplified overview of the interplay between NAD^+^, homocysteine and folate metabolism Multiple pathways contribute to maintaining NAD homeostasis. The *de novo* pathway comprises synthesis from tryptophan via quinolinic acid, whereas the Preiss-Handler pathway synthesizes NAD^+^ from nicotinic acid. They converge at the generation of nicotinic acid mononucleotide (NAMN), which is further converted to nicotinic acid adenine dinucleotide (NAAD) and finally to NAD^+^. NAD^+^ consuming enzymes (e.g., sirtuins, PARPs and NAD^+^ glycohydrolases) degrade NAD^+^ and generate nicotinamide (NAM) in the process. The salvage pathway recycles nicotinamide to NAD^+^ via nicotinamide mononucleotide (NMN). Nicotinamide riboside (NR) is incorporated into NAD metabolism by conversion to either NMN or NAM. NAM can also undergo methyl-dependent conversion to methyl-nicotinamide (MeNAM) where the methyl group is provided by S-adenosylmethionine (SAM), generating S-adenosylhomocysteine (SAH) in the homocysteine cycle. Re-methylation of homocysteine to methionine requires a methyl group donated by methyl-tetrahydrofolate (methyl-THF). Synthesis of methyl-THF is achieved through conversion of 5,10-methylenetetrahydrofolate (5,10-MTHF), catalyzed by methylenetetrahydrofolate reductase (MTHFR). MeNAM and its oxidized metabolites (Me-2-PY/Me-4-PY) are excreted in the urine. The figure is based on multiple sources,^13^^,^^19^^,^^20^^,^^21^^,^^22^^,^^23^^,^^24^^,^^25^ and some intermediary steps, enzymes and compounds are not shown for simplicity. Additional abbreviations: Me-2-PY: N-methyl-2-pyridone-5-carboxamide; Me-4-PY: N-methyl-4-pyridone-5-carboxamide; MS: methionine synthase; NNMT: nicotinamide N-methyltransferase; THF: tetrahydrofolate.

Although NR is generally considered to be safe and to cause only few, if any, adverse effects,^11^ concerns have been raised that augmenting NAD-metabolism may compromise methylation reactions because of increased production and methylation-dependent breakdown of nicotinamide (NAM).^12^ NAD^+^ consuming reactions include the cleavage of NAM, which can either be recycled for NAD biosynthesis or excreted, a process involving methylation of NAM. Here, nicotinamide-N-methyltransferase (NNMT) transfers a methyl group from the universal methyl donor S-adenosylmethionine (SAM) to NAM, producing methyl-nicotinamide (MeNAM) and S-adenosylhomocysteine (SAH), which is further converted to homocysteine as part of the homocysteine-methionine cycle^13^ (Figure 1). Thus, it has been postulated that high dose NAD precursor supplementation may lead to depletion of methyl groups because of increased MeNAM generation and overconsumption of SAM, which could result in impairment of methylation homeostasis and elevated homocysteine levels (Figure 1).^12^ Experiments in rats using high doses of NAM or nicotinic acid (NA) over a course of several weeks have provided some support for this hypothesis. NAM and NA increased the levels of MeNAM (and its oxidized metabolites N-methyl-2-pyridone-5-carboxamide (Me-2-PY) and N-methyl-4-pyridone-5-carboxamide (Me-4-PY)), and induced hepatic steatosis, which could be rescued by methyl group donors such as choline and betaine.^14^^,^^15^ NAM treatment decreased hepatic SAM levels when given to animals fed on a low methionine diet lacking choline^16^ and induced a dose dependent decrease in the level of global DNA methylation in the liver,^17^ whereas NA increased plasma and urine homocysteine concentrations.^18^

NAD supplementation therapy in humans has also been shown to increase the levels of MeNAM, Me-2-PY and Me-4-PY.^10^^,^^26^ If this results in depletion of the methyl group pool, it could compromise several critical processes that depend on methyl group availability, including DNA methylation. If this was the case, it would be imperative that appropriate biomarkers of methylation homeostasis are monitored and, if necessary, methyl group donors administered as part of the treatment regime. Because human data are lacking, however, and studies in animals generally used much higher doses than the ones commonly administered to humans and/or stress-inducing conditions such as methyl group poor diets, this pertinent question remains unaddressed.

**Figure 2:**
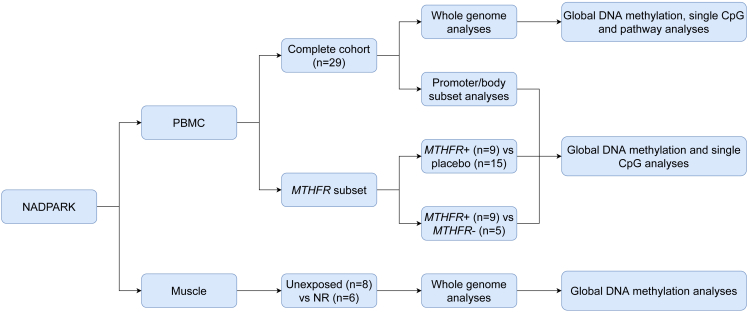
Analysis overview First, we investigated whether NR influenced the global levels or genomic distribution of DNA methylation at the level of single CpGs and biological pathways. A separate analysis was restricted to CpGs in gene promoters or bodies as they have been shown to be the least and most variable over time, respectively.^29^ Then, we assessed whether NR altered DNA methylation specifically in individuals harboring variants in the *MTHFR* gene that are known to decrease the activity of the methyltetrahydrofolate reductase enzyme (Figure 1).^30^ Finally, we investigated the effect of NR on global DNA methylation levels in available muscle samples. *MTHFR*+: Individuals in the NR group with either the C677T or the A1298C *MTHFR* mutation in at least one allele; *MTHFR*-: Individuals in the NR group without the C677T and A1298C *MTHFR* mutations.

We hypothesized that if NAD-supplementation therapy impairs methylation metabolism in humans, this would be reflected in changes in the levels and distribution of DNA methylation. The homeostasis of DNA methylation is reliant on a constant supply of methyl groups from the methionine-homocysteine cycle,^27^ and low availability of methyl group donors, such as folate, has been associated with global DNA hypomethylation in mice.^28^ To test our hypothesis, we harnessed biological material and data from the NADPARK study, a phase I clinical trial comparing NR 1,000 mg daily to placebo in 30 individuals with newly diagnosed, treatment naïve PD.^10^ In the NADPARK trial, NR-recipients showed increased levels of MeNAM and/or its degradation products Me-2-PY/Me-4-PY in blood cells, muscle and the cerebrospinal fluid.^10^ On the other hand, no change was observed in the levels of SAM, SAH, homocysteine, or adenosine, suggesting SAM consumption was modest. To assess whether DNA methylation homeostasis was affected in the NADPARK participants, we performed a genome-wide analysis of DNA methylation. A flowchart summarizing our analyses is shown in Figure 2.

## Results

First, we investigated whether NR influenced the global levels of DNA methylation, or its genomic distribution at the level of single CpGs and biological pathways. After quality filtering, a total of 661,049 CpGs were assessed in PBMC samples of 29 individuals, 14 in the NR group and 15 in the placebo group. We identified 28,451 non-variable CpGs, accounting for 4.3% of the total number. Because of the relatively low number we chose to retain these CpGs in the dataset. Analyses followed a paired design, comparing samples taken at the end of the trial (day 30) to baseline per individual.

**Figure 3:**
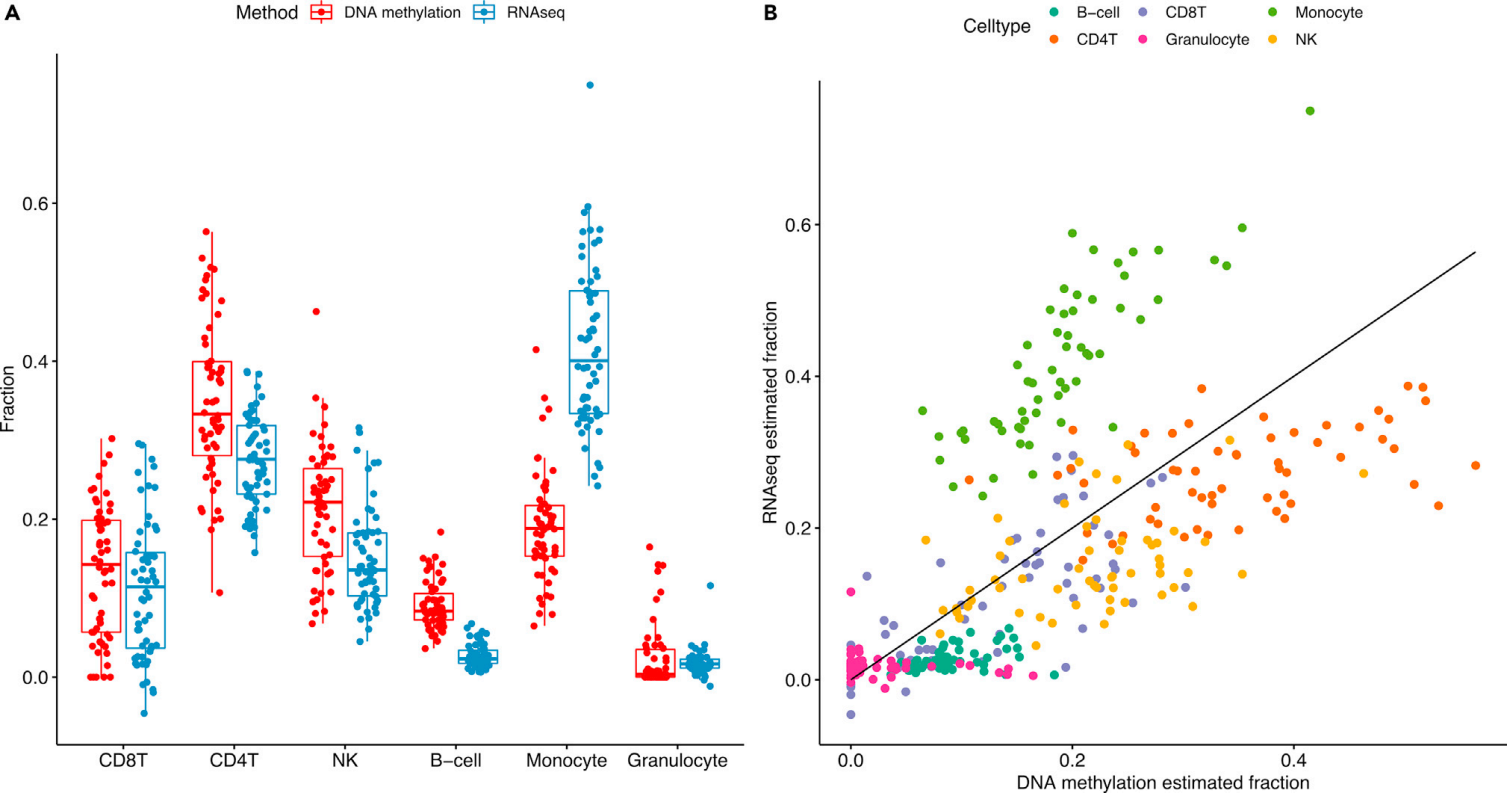
Comparing DNA methylation and RNAseq based cell type estimates (A) Boxplot of DNA methylation (red) and RNAseq (blue) based cell type estimates as measured by fraction of total cell count. The boxes display median and interquartile range. (B) Plot of cell type estimates per individual. Points represent the estimated fraction based on DNA methylation (x-axis) and RNAseq (y-axis) for a specific individual and cell type. RNAseq based cell type estimates have been previously published.^10^ CD8T: CD8-positive T-cells; CD4T: CD4-positive T-cells; NK: Natural killer cells.

Because different types of white blood cells have distinct DNA methylation patterns, changes in cell composition between the visits could substantially bias the results of our analyses.^31^ To account for this effect, we estimated cell type composition in our samples from DNA methylation data and compared them to our previously published estimates based on RNAseq data from the same PBMC samples.^10^ Although the methods showed comparable estimates at the group level (Figure 3A), we found substantial differences at the individual level (Figure 3B). Because of this, surrogate variables were used instead to account for differences in cell type composition between the samples.

### NR treatment does not alter global DNA methylation

**Figure 4:**
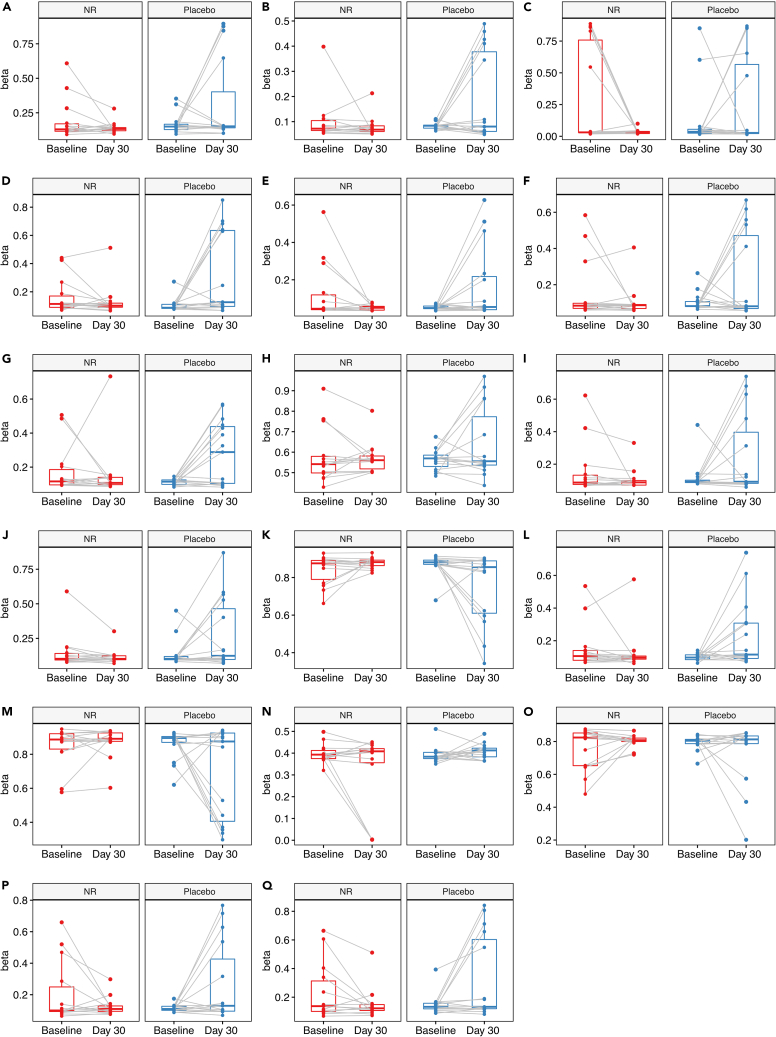
Analysis of methylation variance Plots showing the methylated fraction of CpGs (beta) at baseline and day 30 for the 17 CpGs with statistically significant changes in methylation variance. Points connected with a gray line represent the same individual at baseline and day 30. The boxes display median and interquartile range. (A) cg16826718. (B) cg06895197. (C) cg06895197. (D) cg11521799. (E) cg14459930. (F) cg27507339. (G) cg16877339. (H) cg07866632. (I) cg14451561. (J) cg03558326. (K) cg19507893. (L) cg00222056. (M) cg13536076. (N) cg21223075. (O) cg22746566. (P) cg22746566. (Q) cg18109941.

NR treatment did not influence global DNA methylation levels in PBMCs (p = 0.18). Single CpG analyses similarly revealed no significant effect of NR on DNA methylation profile (Table S1). Pathway enrichment analysis on the results of the single CpG analysis revealed significant enrichment in 10 pathways after correcting for multiple testing (Table 1). However, analysis of the transcriptomic data from the same samples showed no significant expression changes in these pathways, irrespective of direction (Table S2). Analysis of methylation variance revealed statistically significant changes for 17 individual CpGs in the NR group compared to the placebo group. For these CpGs, variance was generally decreased in the NR group. (Table 2 and Figure 4).

**Table 1:** Pathway analysis

ID	Description	Size	p-value	Adjusted p
GO:0046015	regulation of transcription by glucose	10	3.55E-07	0.0029
GO:0090661	box H/ACA telomerase RNP complex	11	2.04E-06	0.0065
GO:0031429	box H/ACA snoRNP complex	13	2.41E-06	0.0065
GO:0032011	ARF protein signal transduction	19	4.38E-06	0.0070
GO:0032012	regulation of ARF protein signal transduction	19	4.38E-06	0.0070
GO:0008327	methyl-CpG binding	29	7.68E-06	0.0103
GO:0061511	centriole elongation	10	1.42E-05	0.0163
GO:0007549	dosage compensation	22	2.12E-05	0.0189
GO:0009048	dosage compensation by inactivation of X chromosome	20	2.12E-05	0.0189
GO:0034244	negative regulation of transcription elongation from RNA polymerase II promoter	17	3.04E-05	0.0244

Significantly enriched pathways after application of FDR-correction. ID: pathway Gene Ontology ID; Size: number of genes in pathway; Adjusted p: FDR-corrected p value.

**Table 2:** Analysis of methylation variance

CpG	Gene	Group	Location	logFC	Average M	Average beta	p-value	Adjusted p
cg16826718	HRK	3′UTR	North shore	−1.798	0.869	0.646	2.32E-08	0.008
cg06895197	PHF12	TSS200	North shore	−1.367	0.665	0.613	2.34E-08	0.008
cg09672912	–	–	–	−3.484	1.714	0.766	1.02E-07	0.022
cg11521799	SNRPB	TSS200	South shore	−1.591	0.928	0.655	1.51E-07	0.025
cg14459930	JKAMP/L3HYPDH	5′UTR/1stExon/TSS1500/body	South shore	−1.907	0.892	0.650	1.92E-07	0.025
cg27507339	SLCO5A1	5′UTR/TSS1500	Island	−1.627	0.969	0.662	4.30E-07	0.036
cg16877339	–	–	–	−1.504	0.769	0.630	4.38E-07	0.036
cg07866632	CCDC101	5′UTR	–	−1.071	0.605	0.603	5.13E-07	0.036
cg14451561	AMACR/C1QTNF3	TSS1500/body	South shore	−1.442	0.891	0.650	5.35E-07	0.036
cg03558326	C14orf167/DHRS4	Body/1stExon	Island	−1.300	0.823	0.639	5.48E-07	0.036
cg19507893	–	–	North shore	−1.029	0.615	0.605	6.49E-07	0.036
cg00222056	TMEM14A	TSS1500	North shore	−1.398	0.747	0.627	6.55E-07	0.036
cg13536076	ERICH6-AS1	Body	South shelf	−1.386	0.935	0.657	8.07E-07	0.041
cg21223075	BHLHE40	TSS1500	Island	1.996	0.630	0.608	8.99E-07	0.042
cg22746566	C2CD2	5′UTR/body	–	−1.063	0.546	0.593	9.93E-07	0.044
cg27587195	MCC	5′UTR/1stExon	Island	−1.705	0.892	0.650	1.18E-06	0.048
cg18109941	PLXDC2	1stExon/5′UTR	Island	−1.599	1.021	0.670	1.24E-06	0.048

The table displays results from the single CpG analysis of methylation variance in the complete dataset. Only the 17 CpGs with statistically significant differences in methylation variance after correcting for multiple testing are shown. logFC: log-fold change in methylation variance. TSS1500/TSS200: promoter regions 1500/200 base pairs upstream from the transcription start site. Adjusted p: FDR corrected p values. Gene/location mappings are from the Illumina manifest (Infinium MethylationEPIC v1.0 B5) which in turn is based on the USCS database.^32^

To assess the effect of NR on DNA methylation in a different tissue, we analyzed available muscle biopsy samples. Unfortunately, because of the scarcity of this material, sufficient muscle tissue was only available from a subgroup of study participants from either baseline visit or visit after treatment, and only from two study participants from both visits. We therefore performed an unpaired comparison of global DNA methylation levels between 6 NR recipients and 8 (age-matched) individuals who had not been exposed to NR. This comparison revealed no difference between the groups (p = 0.95, Figure S3A). Moreover, paired comparison of global DNA methylation levels in the two paired samples revealed no significant change (p = 0.24, Figure S3B).

### NR treatment does not influence global DNA methylation in promoters or gene bodies

There is evidence suggesting that CpGs localized in the gene body are generally more prone to variation of their methylation status over time compared to promoter CpGs.^29^ Thus, we hypothesized that if NR has only a subtle effect on DNA methylation, this may not be detectable on a genome-wide scale, but may emerge when restricting the analysis to the gene body CpGs alone. In this scenario, consequently the methylation status of promoter regions would be expected to remain comparatively unchanged. To assess this, we performed two additional analyses restricted to CpGs in promoter regions or gene bodies.

**Figure 5:**
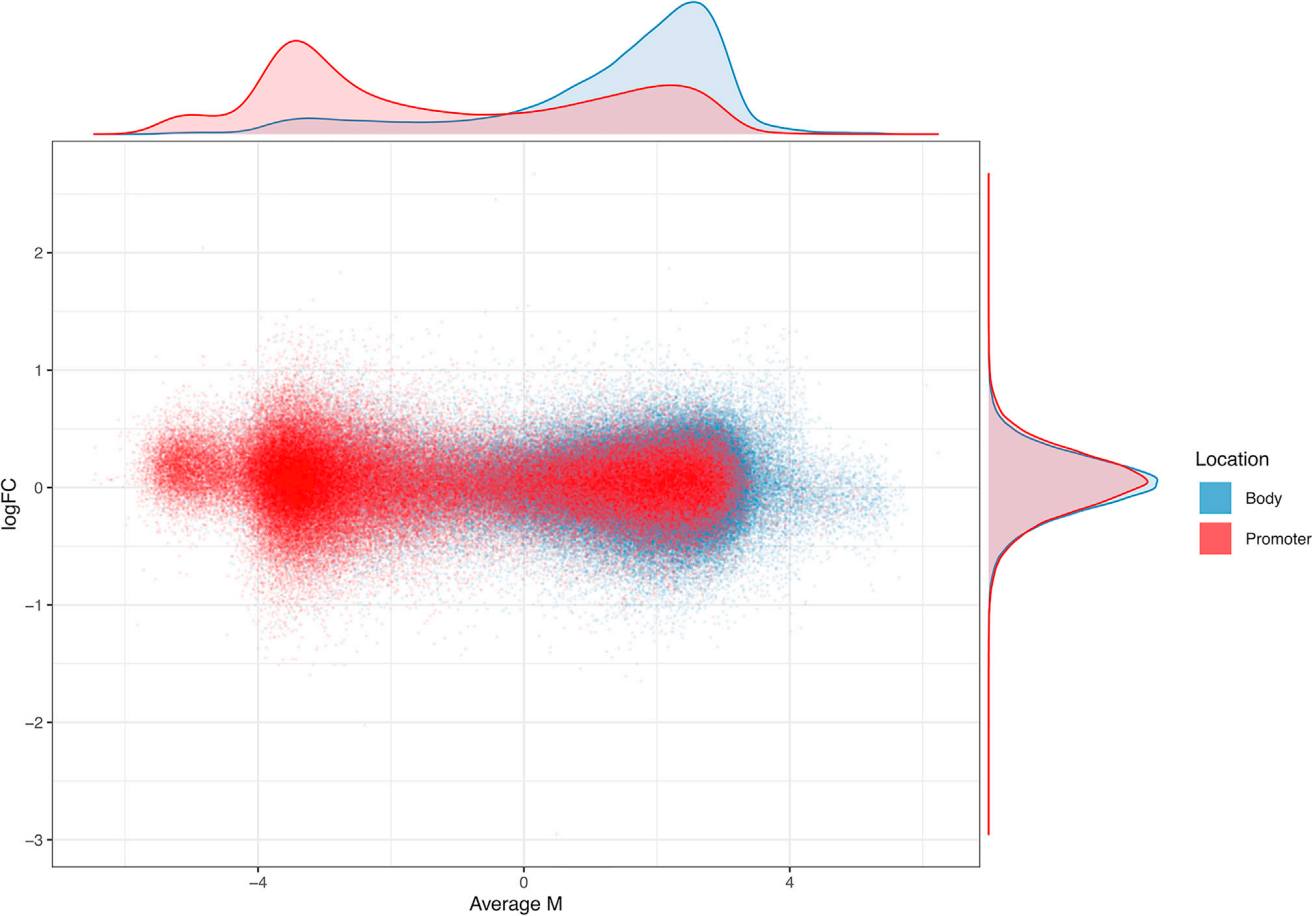
Gene body and promoter CpG methylation status is unaltered by NR Each dot represents one CpG, and the average methylation across all samples (Average M) and the log-fold change between NR and placebo (logFC) are shown. M-values are logarithmic transformations of methylation fraction (beta) values, thus, M-values of −2 and 2 translate to a methylation fraction (beta) of 0.2 and 0.8, respectively. Red dots are promoter CpGs, and blue dots are gene body CpGs. Density plots along their representative axis are shown outside the main plot.

We identified 227,613 CpGs located in gene bodies, and 104,900 CpGs located in promoter regions. Restricting our analyses to these subsets detected no significant change in overall levels of methylation across all gene body (p = 0.21) or promoter (p = 0.12) CpGs, or differential methylation of specific CpGs. As an incidental observation, we noted that gene body CpGs in our sample tended to be much more methylated than promoter CpGs (for all individuals, irrespective of NR treatment, see Figure 5).

### *MTHFR* variation is not associated with NR-induced changes in DNA methylation

Finally, we assessed whether NR altered DNA methylation specifically in individuals harboring variants in the *MTHFR* gene that are known to decrease the activity of methyltetrahydrofolate reductase (Figure 1). The folate cycle is an important donor of methyl groups, facilitating the remethylation of homocysteine to methionine which, after conversion to SAM, in turn serves as a central methyl group donor in mammalian cells for a wide variety of processes, including DNA methylation.^27^ The MTHFR enzyme serves an important role by catalyzing the conversion of 5,10-MTHF to methyl-THF, and is therefore, by extension, essential for replenishing the levels of methionine and SAM^30^ (Figure 1). The common *MTHFR* variants C677T (p.A222V, rs1801133) and A1298C (p.E429A, rs1801131) have been shown to decrease the enzymatic function of MTHFR.^30^ It is therefore possible that individuals carrying either of these variants may be more susceptible to methyl group depletion. To test this hypothesis, we stratified our analyses according to *MTHFR* genotype. Nine individuals in the NR group were heterozygous for either the C677T mutation (n = 2) or the A1298C mutation (n = 7) in the *MTHFR* gene (Table S3). We performed a differential DNA methylation analysis comparing these individuals to NR recipients without *MTHFR* variation (n = 5) or to the placebo group (n = 15). There were no significant differences with regard to global DNA methylation or methylation status of individual CpGs (Tables S4 and S5). Two CpGs showed statistically significant changes in methylation variance when comparing individuals with and without *MTHFR* variation in the NR group (cg16026114 [adjusted p = 0.016] and cg07573057 [adjusted p = 0.016], Table S5 and Figure S4). For both CpGs, this change consisted of increased variance in the wild type group, and is therefore unlikely to reflect an NR-specific effect.

**Figure 6:**
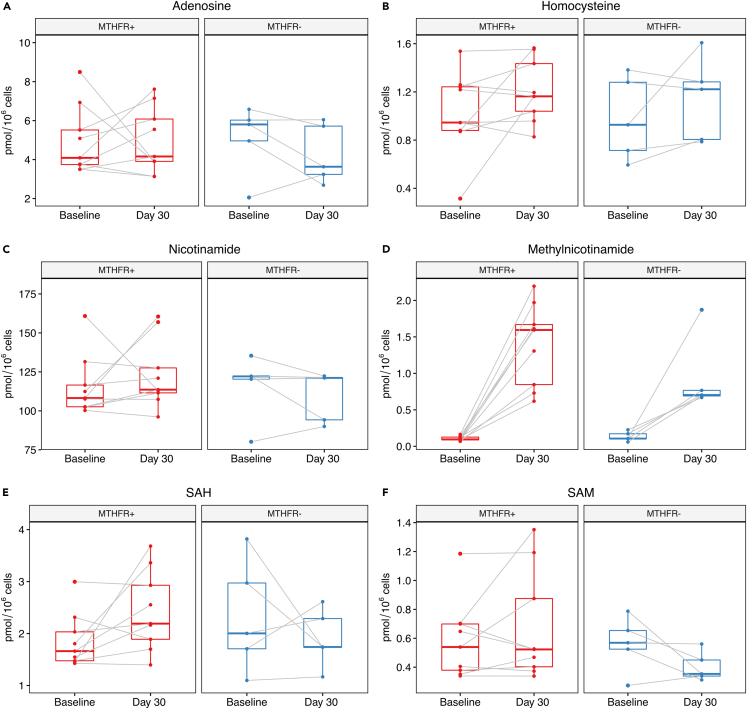
Impact of *MTHFR* genotype on methylation-relevant key metabolites in response to NR in PBMCs Points connected with a gray line represent the same individual at baseline and day 30. The boxes display median and interquartile range. The effect of *MTHFR* genotype and NR on key metabolites were investigated using a two-way repeated measures ANOVA. p-values for the interaction between MTHFR genotype and NR treatment. (A) Adenosine p = 0.52. (B) Homocysteine p = 0.89. (C) Nicotinamide p = 0.36. (D) Methylnicotinamide p = 0.14. (E) SAH p = 0.10. (F) SAM p = 0.086. MTHFR+: individuals heterozygous for either the C677T or A1298C mutation; MTHFR-: individuals with wild type *MTHFR* status. Metabolomics data are taken from Brakedal et al.^10^ and reanalyzed according to *MTHFR* status in this study. Measurements stratified by *MTHFR* genotype can be found in Data S1.

**Figure 7:**
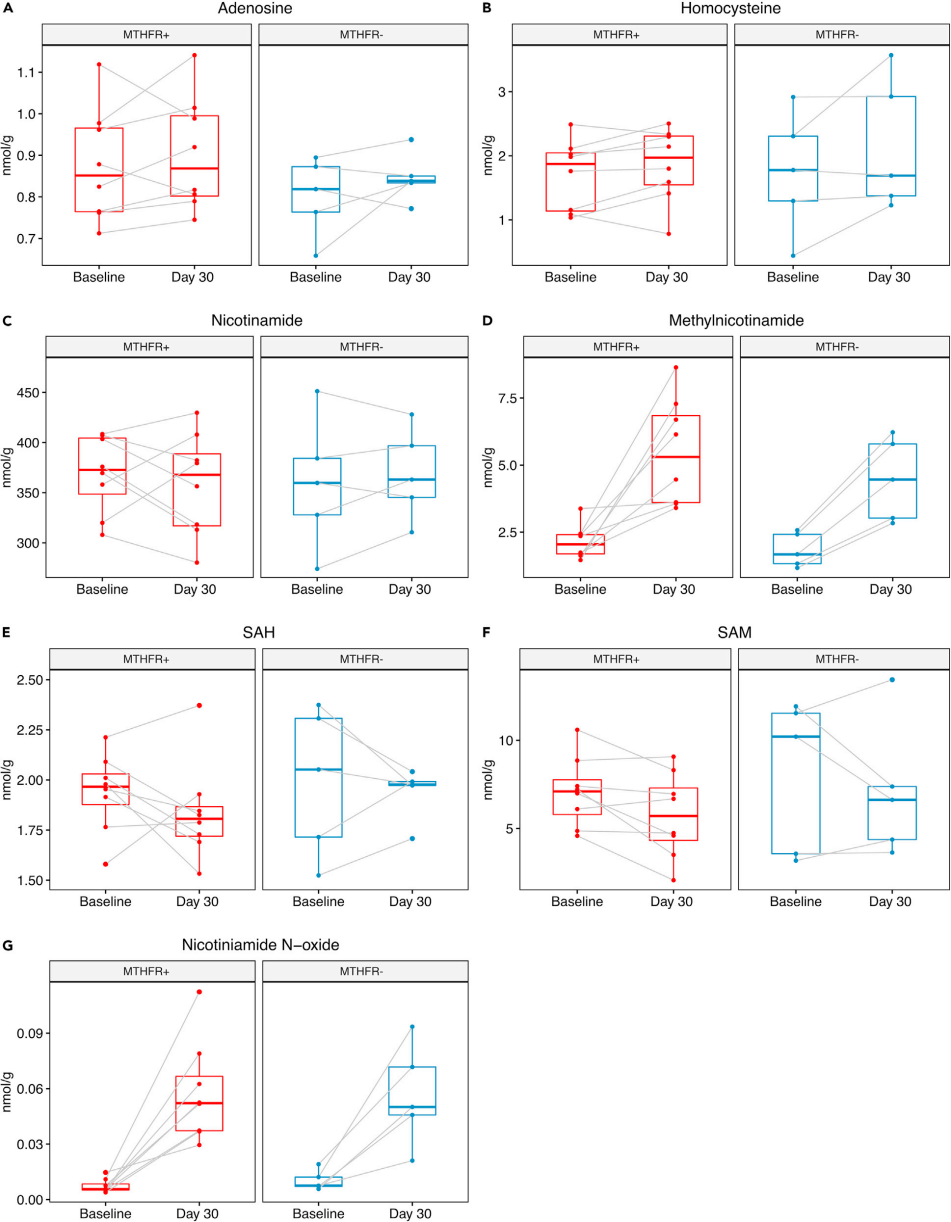
Impact of *MTHFR* genotype on methylation-relevant key metabolites in response to NR in muscle Points connected with a gray line represent the same individual at baseline and day 30. The boxes display median and interquartile range. The effect of *MTHFR* genotype and NR on key metabolites were investigated using a two-way repeated measures ANOVA. p-values for the interaction between MTHFR genotype and NR treatment. (A) Adenosine p = 0.75. (B) Homocysteine p = 0.30. (C) Nicotinamide p = 0.42. (D) Methylnicotinamide p = 0.52. (E) SAH p = 0.77. (F) SAM p = 0.78. (G) Nicotinamide N-oxide p = 0.78. MTHFR+: individuals heterozygous for either the C677T or A1298C mutation; MTHFR-: individuals with wild type *MTHFR* status. Metabolomics data are taken from Brakedal et al.^10^ and reanalyzed according to *MTHFR* status in this study. Measurements stratified by *MTHFR* genotype can be found in Data S1.

We also investigated whether there was a combined effect of NR treatment and *MTHFR* variation on the level of several key metabolites in PBMCs (i.e., adenosine, homocysteine, NAM, MeNAM, SAH, SAM) by performing a two-way repeated measures ANOVA and looking at the interaction effect between MTHFR genotype and NR treatment (before and after). We did not detect any statistically significant effects of MTHFR genotype on metabolites in either PBMC or muscle (see Figures 6 and 7, and Table S6).

## Discussion

We show that NAD-replenishment therapy by oral supplementation with 1,000 mg NR daily for 30 days has no influence on global levels or genome-wide distribution of DNA methylation. Considered together with the fact that the same treatment had no effect on the levels of methylation-relevant metabolites SAM, SAH, adenosine, or homocysteine,^10^ our findings suggest that at least short-term treatment with clinically approved doses of NR has no significant impact on methylation homeostasis in adult humans.

These findings are not entirely unexpected. A normal diet is expected to provide sufficient one-carbon metabolism nutrients to replenish the SAM-pool even at high consumption rates. In line with this notion, it has been shown that physiological variation in the dietary intake of one-carbon metabolism nutrients does not influence DNA methylation in humans.^33^ Currently, the only evidence that NAD replenishment could lead to methylation depletion derives from studies in which animals were given very high doses of NAD precursors commonly combined with stress conditions such as dietary deprivation of methyl-donors.^11^^,^^12^^,^^13^^,^^14^^,^^15^

Our differential methylation analyses detected no significant changes at the level of single CpGs, promoters or gene bodies. Pathway analyses showed significant enrichment in ten biological processes, mostly related to transcriptional regulation. However, pathway enrichment analyses should be interpreted with caution in the absence of significant changes in individual CpGs or genes. Moreover, these changes were not associated with altered gene expression in the same pathways. Thus, although these results raise the possibility that NR supplementation may be associated with rare changes in specific DNA methylation sites, our data suggest that this cannot be attributed to a general effect of NR on DNA methylation per se.

Analysis of methylation variance detected 17 CpGs with significantly altered variability when comparing the NR and placebo groups. Of interest, 16 of these 17 CpGs showed reduced variability in the NR group (Table 2). Considering the individual CpG plots (Figure 4), it is evident that for most of these CpGs the difference can be ascribed to increased variability after 30 days in the placebo group, as opposed to a reduction in variability in the NR group. As such, we propose that these results are more likely to be false positives.

Common *MTHFR* variations were not associated with susceptibility to NR-induced changes in DNA methylation or methylation relevant metabolites. Although homocysteinemia has only been conclusively established in individuals with biallelic *MTHFR* variants,^34^^,^^35^ heterozygosity for the C677T or A1298C variants has also been shown to strongly reduce the enzymatic activity of MTHFR.^36^ Because the C677T and A1298C variants are very common, with minor allele frequencies of 0.34 and 0.32, respectively, in the non-Finnish European population (gnomAD v2.1.1),^37^ and given the increasingly widespread use of NR and other NAD replenishing compounds, our study provides important data supporting their safety. However, our findings cannot exclude the possibility that individuals with biallelic mutations may be susceptible to methyl group depletion on exposure to NAD replenishment therapies.

In conclusion, our study provides evidence that NAD replenishment therapy with high-dose NR does not compromise DNA methylation homeostasis and does not alter the global DNA methylation levels and landscape in human blood cells. Based on these results, we propose that supplementation with methyl donors or monitoring of methylation metabolism is not required, at least for short-term NR intake. Longer studies, and measurements in other clinically testable human tissues are warranted to confirm whether this is the case also with long-term use of NR and other NAD-precursors.

### Limitations of the study

A limitation of our study is the relatively short timeframe of exposure (30 days). Based on our data, we cannot exclude that a long-term exposure to NR may over time incrementally impact methylation homeostasis, including DNA methylation. However, the blood concentration of methylated degradation products of NAD metabolism (MeNAM, Me-2-PY, Me-4-PY) rises steeply within only a few hours after oral intake of NR,^19^ and was found to be substantially increased in all NR-recipients at the end of the study.^10^ It is therefore safe to assume that the production and excretion of these metabolites was at a steady state of high turnover for the duration of the study. Given the high turnover rate of PBMCs, most would have been completely replaced during the study. Monocytes and granulocytes are relatively short lived in the bloodstream (approx. 1 and 5.4 days on average, respectively),^38^^,^^39^ whereas T-cells are more long lived (turnover times ranging from 2–6 weeks).^40^^,^^41^ Given this, and the dynamic nature of DNA methylation, we deem it highly likely that a biologically significant NR-induced methyl group depletion would have manifested within the time frame of our study. Still, data from longer studies are needed to confidently exclude this possibility. It should also be stressed that our findings in PBMCs do not necessarily reflect the state of other tissues and organs.

All participants in the study had PD, but were newly diagnosed and drug-naïve. They received no additional treatment for PD during the course of the study, so we do not believe this impacts the applicability of our results to the general population.

The number of female participants in the study was low, likely related to the higher risk of PD in males,^42^ and the proportion of females were higher in the NR group than the placebo (4 of 14 vs 1 of 15, see Table S7). We are not aware of any evidence suggesting that the effect of an NAD precursor on DNA methylation and other methylation reactions would be different in males and females, and therefore do not believe this affects the interpretation of our results.

## STAR★Methods

### Key resources table

**Table:** 

REAGENT or RESOURCE	SOURCE	IDENTIFIER
**Critical commercial assays**		

MethylationEPIC BeadChip v1.0	Illumina	Cat#WG-317

**Deposited data**		

Metabolomic data, stratified by *MTHFR* genotype	This paper	Data S1 (data supporting Figures 6 and 7)
DNA methylation data	This paper	https://git.app.uib.no/neuromics/nadpark-methylation
RNAseq data	Brakedal et al.^10^	https://git.app.uib.no/neuromics/nadpark
Original code	This paper	https://git.app.uib.no/neuromics/nadpark-methylation and https://git.app.uib.no/neuromics/nadpark

**Software and algorithms**		

R v3.6.3 and v4.1.1	R core team	RRID:SCR_001905; https://www.r-project.org/
R package minfi (v1.28.4)	Fortin JP et al.^43^	RRID:SCR_012830; https://www.bioconductor.org/packages/release/bioc/html/minfi.html
R package missMethyl (v1.26.1)	Phipson B et al.^44^	https://bioconductor.org/packages/release/bioc/html/missMethyl.html
R package sva (v3.40.0)	Leek JT et al.^45^	RRID:SCR_002155; https://bioconductor.org/packages/release/bioc/html/sva.html
R package limma (v3.48.3)	Ritchie ME et al.^46^	RRID:SCR_010943; https://bioconductor.org/packages/release/bioc/html/limma.html
R package methylGSA (v1.10.0)	Ren X et al.^47^	https://bioconductor.org/packages/release/bioc/html/methylGSA.html
R package fgsea (v1.18.0)	Korotkevich G et al.^48^	RRID:SCR_020938; https://bioconductor.org/packages/release/bioc/html/fgsea.html
HISAT2 (v2.2.1)	Kim D et al.^49^	RRID:SCR_015530; http://daehwankimlab.github.io/hisat2/
Picard Tools	Broad Institute	RRID:SCR_006525; https://broadinstitute.github.io/picard/
GATK (v4.1.9)	McKenna A et al.^50^	RRID:SCR_001876; https://gatk.broadinstitute.org/
ANNOVAR (vJune2020)	Wang K et al.^51^	RRID:SCR_012821; https://annovar.openbioinformatics.org/

**Other**		

GENCODE release 35	Frankish A et al.^52^	RRID:SCR_014966; https://www.gencodegenes.org
Infinium MethylationEPIC v1.0 B5 Manifest File	Illumina	https://support.illumina.com/downloads/infinium-methylationepic-v1-0-product-files.html

### Resource availability

#### Lead contact

Further information and requests for resources should be directed to and will be fulfilled by the lead contact, Charalampos Tzoulis (Charalampos.Tzoulis@uib.no).

#### Materials availability

This study did not generate new unique reagents.

### Experimental model and subject details

#### Cohorts

Peripheral blood mononuclear cell (PBMC) samples were obtained from the NADPARK study, a phase I, randomized, double-blinded clinical trial of NR in PD. In this study, 30 newly-diagnosed and therapy-naïve patients with PD received either oral 1,000 mg NR daily or placebo for a duration of 30 days.^10^ Baseline and 30-day PBMC samples were available from a total of 29 individuals (NR = 14, placebo = 15). Muscle biopsy was performed on the NADPARK participants at both visits, but obtained material was limited due to the invasiveness of the procedure, and most had been already used up in previous analyses.^10^ Remaining muscle samples comprised paired samples from two individuals (i.e., from both visits, total 4 samples) and 10 unpaired samples (i.e., one visit per individual) collectively amounting to a total of 6 samples with exposure to NR for 30 days and 8 baseline samples. Metabolomic data was available from PBMCs (n = 14) and muscle (n = 13) from the NR group of the NADPARK participants, as described.^10^

Subject characteristics are available in Table S7. The research protocol was approved by the Regional Committee for Medical and Health Research Ethics, Western Norway (2018/597). Patients were identified and recruited at the Department of Neurology, Haukeland University Hospital, Norway. Written and informed consent was obtained from all participants from investigators in NADPARK. This study was conducted according to Good Clinical Practice guidelines. The trial is registered at Clinicaltrials.gov, identifier: NCT03816020.

### Method details

#### Methylation analyses

For DNA extraction, each sample was homogenized in 650ul RLT plus buffer using the TissueLyser II at 30 Hz/sec for 3 min (1 cycle). The lysate tubes were then spun down and 250ul lysate from each sample was taken into DNA isolation on a QIAsymphony instrument as per the manufacturers recommended protocol with RNase digestion. Final dilution was made in 100ul volume, and the DNA samples were stored at −80C. 350ul lysate from each sample was taken into RNA isolation on the RNeasy Plus Mini Kit as per manufacturers recommended protocol with DNase digestion. The isolated RNA samples were stored at −80C. DNA-methylation profile was assessed using the Illumina Infinium MethylationEPIC BeadChip Kit. DNA isolation and the methylation chip were run at HudsonAlpha institute of Biotechnology, AL, USA. Data conversion and quality control was performed using R (version 3.6.3) and Bioconductor package *minfi* (version 1.28.4).^43^

Poor performing probes, as defined as having a detection P as computed by *detectionP* in *minfi* package, value >0.01 in 20% or more of the samples, were removed. The remaining data was SWAN normalized using the *missMethyl* (version 1.26.1) R package.^44^ X and Y chromosome CpGs were also removed and not used for subsequent analyses. A principal component and heatmap analysis showed two outlier samples, but because they were from the same individual (at baseline and after 30 days respectively) we did not remove them from the dataset (see Figures S1 and S2).

To map CpGs to either promoter or gene body regions, we used the product files supplied by Illumina (Infinium MethylationEPIC v1.0 B5). CpGs were classified as promoters only if they exclusively mapped to promoter regions (TSS1500 and/or TSS200). Likewise, CpGs were classified as gene body CpGs only if they exclusively mapped to “body” in the product file (thus excluding CpGs mapping to UTR3, UTR5 or 1^st^ exon).

Celltype estimates were calculated using the *estimateCellCounts* from the *minfi* R package on the methylation chip data.^43^ RNA sequencing (RNAseq) based cell type estimates, available from Brakedal et al.,^10^ from the same PBMC samples were generated using the ABsolute Immune Signal (ABIS) deconvolution method.^53^

#### MTHFR genotyping

In order to genotype single nucleotide variants in the *MTHFR* gene, RNAseq raw fastq files from PBMCs corresponding to the first visit were aligned using HISAT2 v2.2.1^49^ against the hg38 human reference genome and subsequently deduplicated using Picard Tools *MarkDuplicates*.^54^ As recommended by GATK/Broad Institute guidelines for RNAseq variant discovery, deduplicated alignments were processed with the *SplitNCigarReads* tool of GATK (version 4.1.9)^50^prior to variant calling. Base quality score recalibration was carried out using the *BaseRecalibrator* and *ApplyBQSR* tools with the optional flag--use-original-qualities. Variant calling was carried out for each individual using *HaplotypeCaller* and restricted to exonic regions as defined in the GENECODE release 35^52^ (with optional flags--dont-use-soft-clipped-bases). Variants were restricted to single nucleotide polymorphisms (SNP) and filtered using the *VariantFiltration* tool with flags--window 35--cluster 3--filter “FS > 30.0”--filter “QD < 2.0”. The resulting variants were annotated using ANNOVAR (version June2020).^51^

### Quantification and statistical analysis

All statistical analyses were performed using R (version 4.1.1)^55^ and utilizing methylation M values unless otherwise specified. To control for known and unknown covariates in the dataset, surrogate variables were calculated and used in subsequent analyses using the *sva* (version 3.40.0) package with standard settings.^45^ For any analysis performed on a subset of individuals from the original cohort, new surrogate variables were generated. Significant surrogate variables were used as covariates for all subsequent analyses either directly (single CpG linear regression) or indirectly (pathway analysis based on single CpG linear regression p-values). To reduce the number of tests, we utilized a method previously described to identify non-variable CpGs in our sample, with non-variable CpGs defined as having <5% range in beta values between the 10^th^ and 90^th^ percentile.^56^ Single CpG and mean global DNA methylation analyses were performed using linear regression and the *lmfit* function from the *limma* (version 3.48.3)^46^ R package. To assess differences in CpG methylation variability, we used the *varfit* function from the *missMethyl* (version 1.26.1)^44^ R package. For muscle samples, differences in global DNA methylation levels were assessed using either a two-sample t-test (all samples) or paired t-test (repeat samples). Pathway analyses were based on uncorrected p-values obtained from the single CpG analyses, and performed using logistic regression and the *methylglm* function from the *methylGSA* (version 1.10.0)^47^ package. Gene Ontology (GO) pathways were used,^57^^,^^58^ and the analysis restricted to pathway sizes between 10 and 200 genes and to CpGs in promoter regions (group = “promoter2”, using CpGs in “TSS1500”, “TSS200”, “1^st^ exon” and “5’UTR” regions). For all analyses, false discovery rate (FDR) correction was applied to p-values to account for multiple tests. Significant pathways resulting from this enrichment analysis were further assessed for enrichment in the already available RNAseq data from the same PBMC samples using both a *directional* and *non-directional* approach employing the *fgsea*(version 1.18.0) R package.^48^ For the *directional* approach, the genes were ranked according to their differential expression statistic. The resulting list was tested for enrichment at the top (overexpression) and the bottom (underexpression). For the *non-directional* enrichment analysis, genes were ranked according to their differential expression-log(p-value).

Comparisons of metabolite measurements in PBMCs and muscle between subjects receiving NR with and without known pathogenic mutations in *MTHFR* were performed using a two-way repeated measures ANOVA, specifically looking at the interaction between MTHFR genotype and time (before and after NR administration).

### Additional resources

The NADPARK trial is registered at Clinicaltrials.gov under the identifier NCT03816020.

## Data Availability

• The data required to reproduce the results presented in this manuscript have been deposited and are publicly available in the Neuromics Group repository https://git.app.uib.no/neuromics/. The DNA methylation matrix is available at https://git.app.uib.no/neuromics/nadpark-methylation. The RNAseq read count matrix is available at https://git.app.uib.no/neuromics/nadpark (NADPARK/RData/txi_Muscle.Rds and NADPARK/RData/txi_PBMC.Rds). Metabolomic data stratified by *MTHFR* genotype is available in Data S1 (data supporting Figures 6 and 7). • All original code has been deposited and is publicly available in the Neuromics Group repository (https://git.app.uib.no/neuromics/nadpark-methylation and https://git.app.uib.no/neuromics/nadpark). • Any additional information required to reanalyze the data reported in this paper is available from the lead contact upon request.

## Supplemental information

Document S1. Figures S1–S4 and Tables S1–S7

Data S1. Raw cell type fraction and metabolite data, related to Figures 3A, 3B, 6, and 7
