# Supplementary material for: Nicotinamide riboside supplementation is not associated with altered methylation homeostasis in Parkinson’s disease
Source: iScience. 2023 Feb 27;26(3):106278. doi: 10.1016/j.isci.2023.106278 (PMC10014306; doi:10.1016/j.isci.2023.106278)
Supplement: Document S1. Figures S1–S4 and Tables S1–S7 [file mmc1.pdf]

iScience, Volume *26*

Supplemental information

Nicotinamide riboside supplementation is not associated with altered methylation homeostasis in Parkinson's disease

Johannes J. Gaare, Christian Dölle, Brage Brakedal, Kim Brügger, Kristoffer Haugarvoll, Gonzalo S. Nido, and Charalampos Tzoulis

Supplemental figures

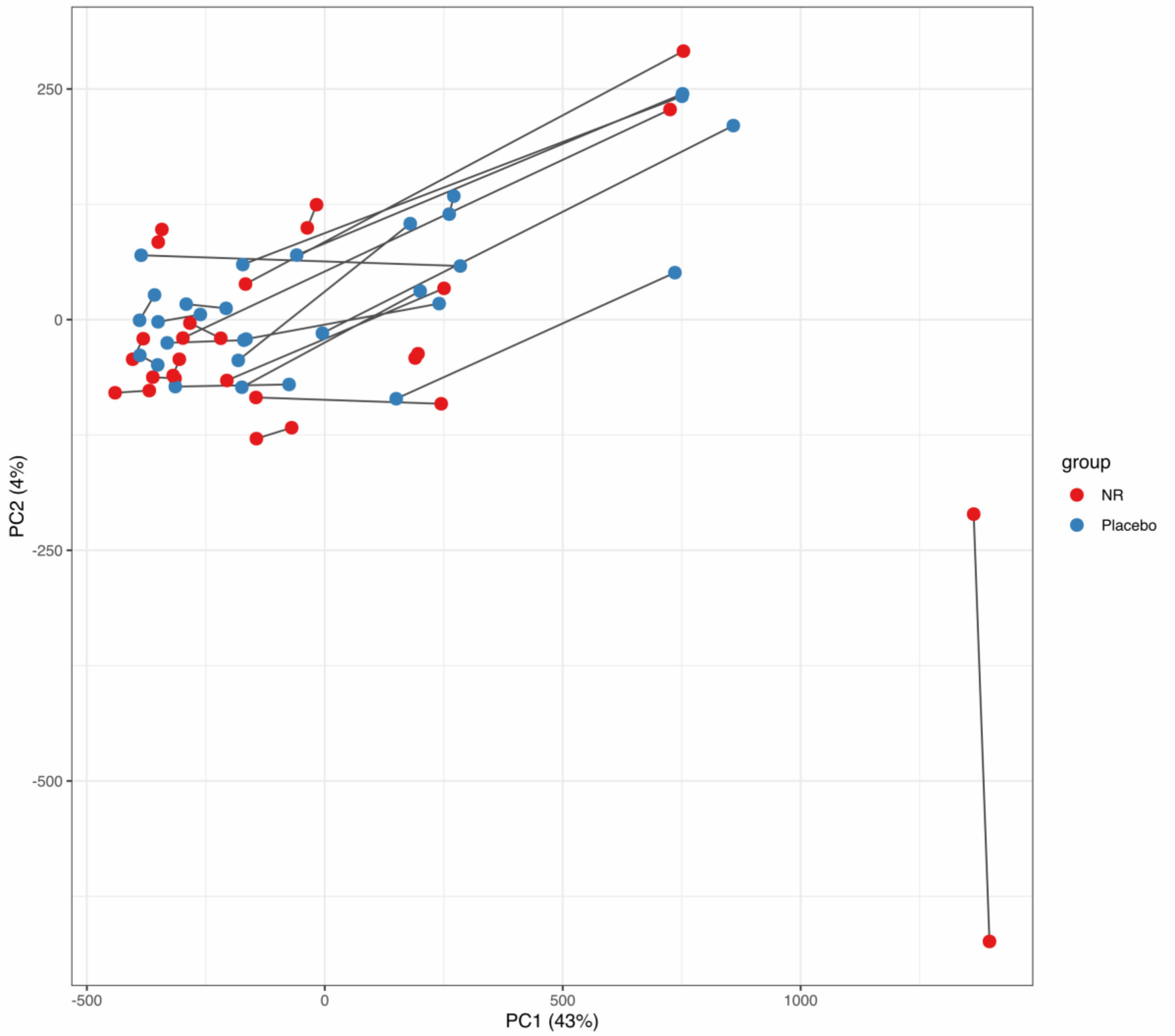

Figure S1. **Principal component analysis. Related to STAR Methods – Method details – Methylation analyses.** Plot showing the first two principal components for the complete methylation dataset. Black lines link the same individual at the different time points (baseline and after 30 days). Percentages in the x- and y-axis description show how much of the total variation is explained by each principal component.

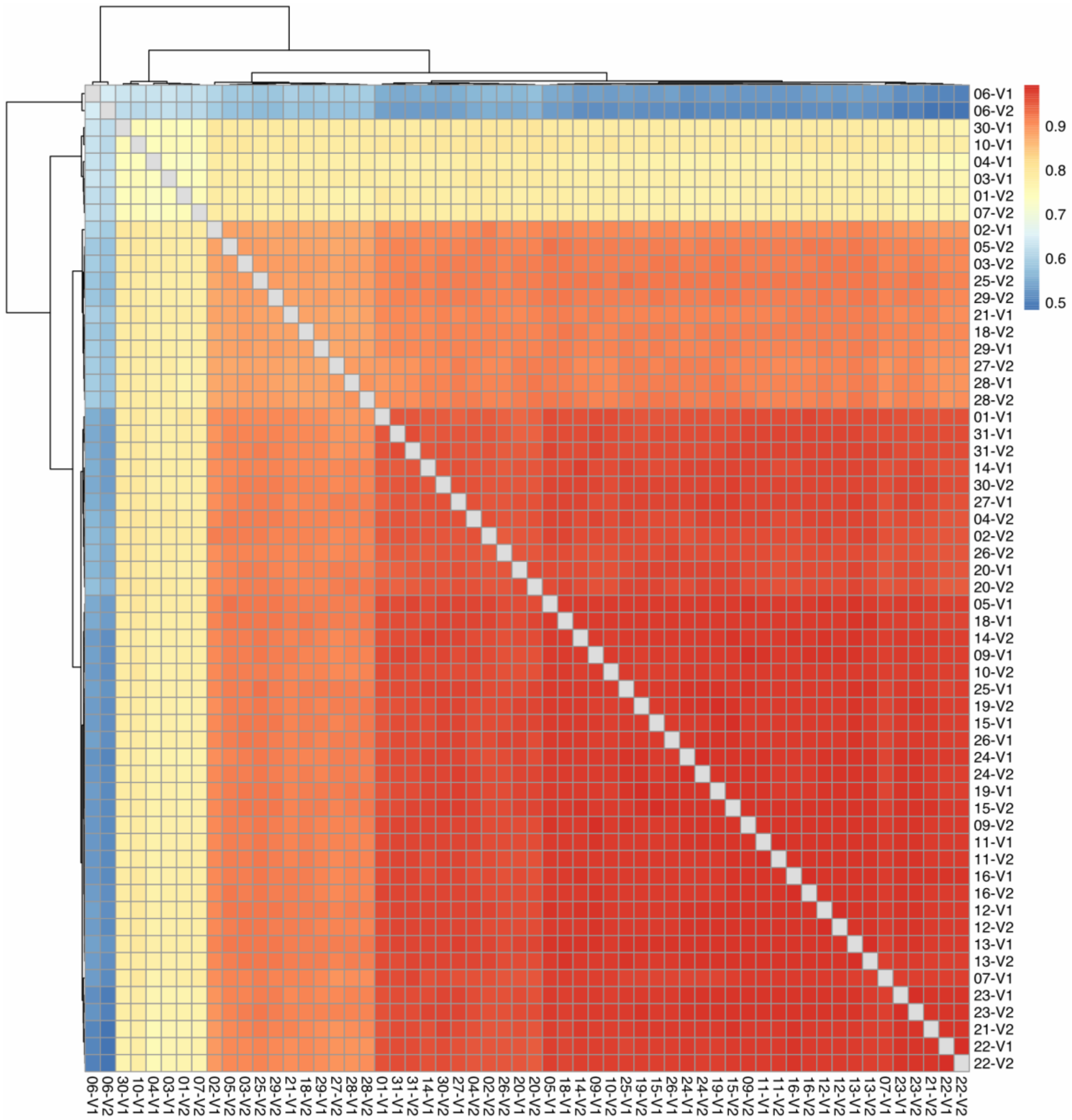

Figure S2: **Heatmap plot showing the degree of similarity and clustering of methylomes of study participants. Related to STAR Methods – Method details – Methylation analyses.** The plot displays the similarities of methylomes for all individuals (numbers 01-31) and visits (V1 [baseline] and V2 [day 30]). The samples are clustered and colored with regard to similarity, with red indicating higher similarity and blue lower similarity. Created using the *pheatmap* R package.

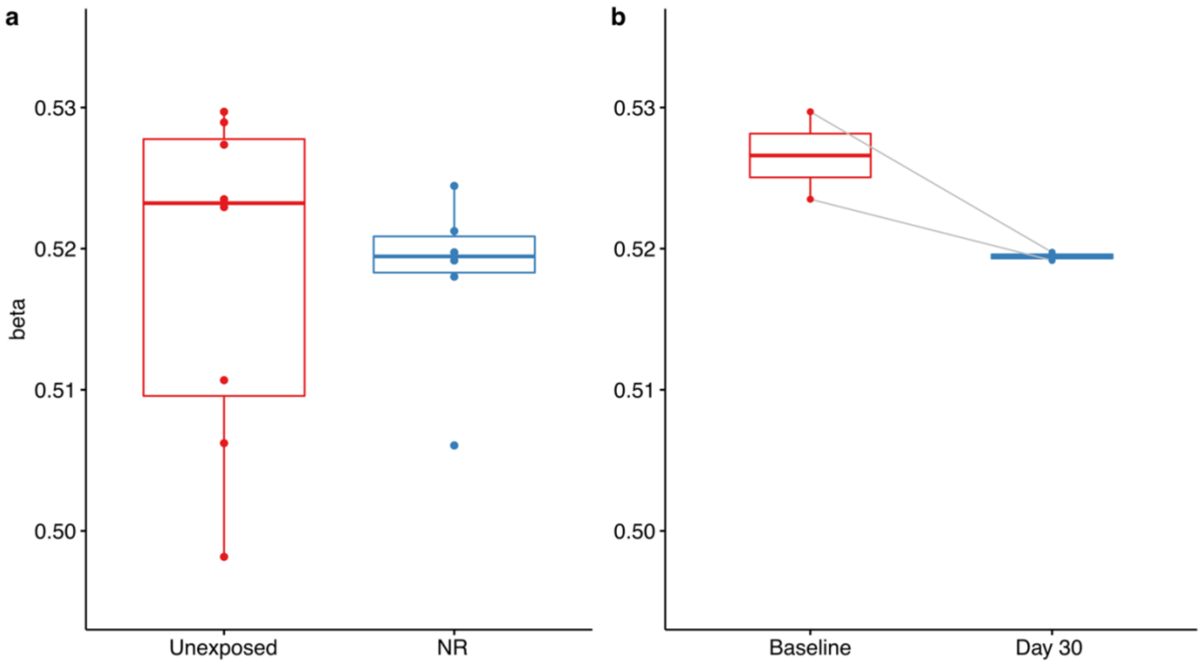

Figure S3. **Global DNA methylation in muscle samples. Related to STAR Methods – Experimental model and subject details - Cohorts** a) Boxplot of all available samples showing the methylated fraction of CpGs (beta) for samples with (blue) and without (red) NR exposure. b) Same as a), showing the available paired samples from two individuals. The boxes display median and interquartile range.

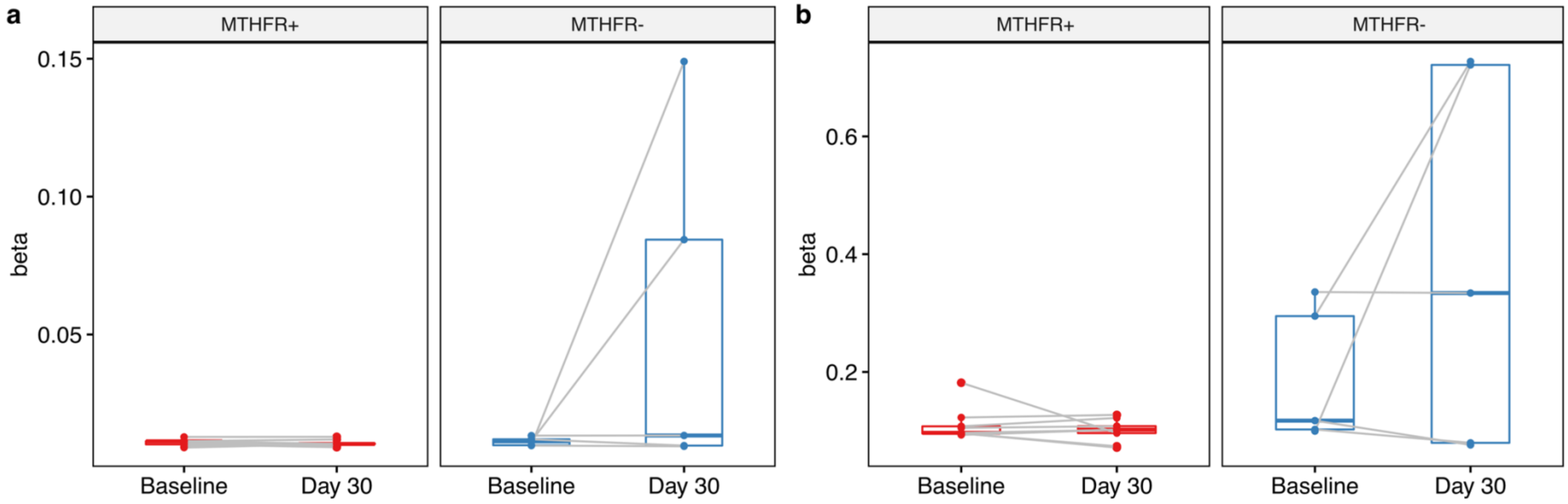

Figure S4. **Change in the methylated fraction of CpGs stratified by *MTHFR* status. Related to STAR Methods – Method details – *MTHFR* genotyping.** Plot showing the change in methylated fraction of CpGs (beta) for **a)** cg16026114 (chr1:232629670-232629672 [hg38]) and **b)** cg07573057 (located in the 5'UTR region of *SNX16*), stratified by *MTHFR* status. MTHFR+: individuals heterozygous for either the C677T or A1298C mutation; MTHFR-: individuals with wild type *MTHFR* status. The boxes display median and interquartile range.

Supplemental tables

Table S1. **Single CpG analysis of changes in methylation in response to NR. Related Table 2.**

CpG	Gene	Group	Location	logFC	Average M	Average beta	p	Adjusted p
cg20709280	OR52B2	TSS1500	-	-1.232	1.006	0.668	2.78E-06	0.823
cg08827322	-	-	-	1.577	-1.418	0.272	8.70E-06	0.823
cg15555250	CCDC88C	Body	-	1.096	-3.478	0.082	1.01E-05	0.823
cg09415959	-	-	-	1.063	-2.635	0.139	1.30E-05	0.823
cg25523462	SUPT4H1	1stExon/5'UTR	Island	1.156	-3.519	0.080	1.53E-05	0.823
cg15529616	-	-	South shore	1.070	-2.580	0.143	2.13E-05	0.823
cg07128503	C16orf74	Body	North shore	-0.921	0.525	0.590	3.35E-05	0.823
cg09343804	MBD2	TSS1500	South shore	-0.892	1.733	0.769	3.43E-05	0.823
cg23530263	NAT14	TSS200	Island	-1.106	-3.574	0.077	3.53E-05	0.823
cg04808988	LHX4	TSS200	Island	0.867	-5.117	0.028	3.61E-05	0.823
cg11852247	STAM2	Body	-	-1.137	2.056	0.806	3.68E-05	0.823
cg07370464	MAP3K14	TSS200	North shore	-0.867	-2.450	0.155	4.08E-05	0.823
ch.11.71909907R	-	-	-	1.278	-2.951	0.115	4.10E-05	0.823
cg00517168	-	-	-	-0.926	2.543	0.854	4.29E-05	0.823
cg23686086	NFIX	3'UTR	Island	0.849	-3.241	0.096	4.40E-05	0.823
cg08130572	FYN	5'UTR/TSS1500	-	0.857	-2.272	0.171	4.60E-05	0.823
cg16286597	MAST3	Body	Island	1.095	-2.281	0.171	4.60E-05	0.823
cg14998310	ABHD17B/C9orf85	TSS1500/1stExon	South shore	0.868	-4.103	0.055	5.91E-05	0.823
cg19496356	ABCB11	Body	-	0.798	1.148	0.689	5.93E-05	0.823
cg12630147	TET1	5'UTR	South shore	0.815	-1.870	0.215	6.27E-05	0.823

Gene/location mappings are from the Illumina manifest (Infinium MethylationEPIC v1.0 B5). logFC: log-fold change, where positive numbers indicate increased methylation in the NR group as compared to placebo, and vice versa. TSS1500/TSS200: promoter regions 1500/200 base pairs upstream from the transcription start site. Adjusted p: FDR-corrected p-value.

Table S2**. Transcriptomic (RNAseq) analysis of pathways enriched in promoter methylation. Related to Table 1.**
The table shows the results from the RNAseq based pathway enrichment analysis performed on the 10 pathways that were significantly enriched in the

**ID**	**Description**	**Directional decreased**		**Directional increased**		**Non-directional**	
		pval	padj	pval	padj	pval	padj
GO:0007549	dosage compensation	0.48	0.69	0.46	0.66	0.48	0.68
GO:0008327	methyl-CpG binding	0.36	0.69	0.33	0.66	0.35	0.68
GO:0009048	dosage compensation by inactivation of X chromosome	0.48	0.69	0.46	0.66	0.48	0.68
GO:0031429	box H/ACA snoRNP complex	0.09	0.64	0.08	0.50	0.08	0.62
GO:0032011	ARF protein signal transduction	0.71	0.79	0.69	0.76	0.71	0.79
GO:0032012	regulation of ARF protein signal transduction	0.71	0.79	0.69	0.76	0.71	0.79
GO:0034244	negative regulation of transcription elongation from RNA polymerase II promoter	0.81	0.81	0.81	0.81	0.81	0.81
GO:0046015	regulation of transcription by glucose	0.29	0.69	0.26	0.65	0.29	0.68
GO:0061511	centriole elongation	0.19	0.64	0.15	0.50	0.19	0.62
GO:0090661	box H/ACA telomerase RNP complex	0.14	0.64	0.15	0.50	0.16	0.62

enrichment analysis of promoter methylation. pval: uncorrected p-values; padj: FDR-corrected p-values.
Transcriptomic data are taken from Brakedal et al[1], and reanalyzed for this study.

Table S3. ***MTHFR* variants in study participants. Related to STAR Methods – Method details – *MTHFR* genotyping**

Participant	Group	AA change	Mutation	Pathogenic variants	Type
02	NR	p.A222V	C677T (rs1801133)	1	Heterozygous
05	NR	p.E429A	A1298C (rs1801131)	1	Heterozygous
10	NR	p.E429A	A1298C (rs1801131)	1	Heterozygous
13	NR	p.E429A	A1298C (rs1801131)	1	Heterozygous
15	NR	p.E429A	A1298C (rs1801131)	1	Heterozygous
20	NR	p.A222V	C677T (rs1801133)	1	Heterozygous
22	NR	p.E429A	A1298C (rs1801131)	1	Heterozygous
28	NR	p.E429A	A1298C (rs1801131)	1	Heterozygous
31	NR	p.E429A	A1298C (rs1801131)	1	Heterozygous
06	NR	-	-	0	Wild type
11	NR	-	-	0	Wild type
19	NR	-	-	0	Wild type
24	NR	-	-	0	Wild type
30	NR	-	-	0	Wild type
01	Placebo	p.E429A	A1298C (rs1801131)	1	Heterozygous
03	Placebo	p.E429A, pA222V	C677T (rs1801133), A1298C (rs1801131)	2	Compound heterozygous
04	Placebo	p.E429A, pA222V	C677T (rs1801133), A1298C (rs1801131)	2	Compound heterozygous
07	Placebo	p.E429A	A1298C (rs1801131)	2	Homozygous
09	Placebo	p.A222V	C677T (rs1801133)	2	Homozygous
12	Placebo	p.E429A	A1298C (rs1801131)	1	Heterozygous
14	Placebo	p.A222V	C677T (rs1801133)	1	Heterozygous
16	Placebo	p.E429A	C677T (rs1801133)	1	Heterozygous
21	Placebo	p.E429A	C677T (rs1801133)	2	Homozygous
23	Placebo	p.A222V	A1298C (rs1801131)	1	Heterozygous
25	Placebo	p.E429A	C677T (rs1801133)	1	Heterozygous
26	Placebo	p.A222V	A1298C (rs1801131)	1	Heterozygous
27	Placebo	p.E429A	C677T (rs1801133)	2	Homozygous
18	Placebo	-	-	0	Wild type
29	Placebo	-	-	0	Wild type

The table shows the distribution of the A1298C and C677T mutations in the NADPARK dataset.

Table S4. **Single CpG analysis of individuals in the NR group with a pathogenic MTHFR mutation vs placebo. Related to STAR Methods – Method details – *MTHFR* genotyping**

Methylation						Methylation variance					
CpG	logFC	Average M	Average beta	p	Adj.p	CpG	logFC	Average M	Average beta	P	Adj.p
cg15555250	1.291	-3.568	0.078	1.48E-05	0.967	cg05174710	2.246	0.611	0.604	1.65E-06	0.509
cg08827322	1.607	-1.428	0.271	1.5E-05	0.967	cg09672912	-3.533	1.808	0.778	2.08E-06	0.509
cg05425666	-1.607	2.191	0.820	2.16E-05	0.967	cg14134054	-2.577	1.343	0.717	3.70E-06	0.509
cg01085995	-1.158	-3.097	0.105	3.76E-05	0.967	cg23700778	-1.758	0.799	0.635	5.64E-06	0.509
cg03417466	-0.973	0.997	0.666	4.32E-05	0.967	cg07866632	-1.075	0.548	0.594	5.74E-06	0.509
cg11138238	-1.356	1.453	0.732	4.75E-05	0.967	cg16877339	-1.612	0.756	0.628	6.97E-06	0.509
cg09415959	1.152	-2.643	0.138	5.67E-05	0.967	cg00889833	1.359	0.602	0.603	6.98E-06	0.509
cg20709280	-1.115	0.993	0.666	5.7E-05	0.967	cg16826718	-1.760	0.853	0.644	7.07E-06	0.509
cg02145101	-1.103	-3.629	0.075	7.16E-05	0.967	cg25405604	1.813	0.694	0.618	8.07E-06	0.509
cg23496755	-1.286	1.658	0.759	7.47E-05	0.967	cg01622772	0.931	0.441	0.576	1.12E-05	0.509

logFC: log-fold change. Adj.p: FDR-corrected p-value.

Table S5. **Single CpG analysis of individuals in the NR group with vs without a pathogenic MTHFR mutation. Related to STAR Methods – Method details – *MTHFR* genotyping**

Methylation						Methylation variance					
CpG	logFC	Average M	Average beta	P	Adj.p	CpG	logFC	Average M	Average beta	P	Adj.p
cg13495347	-1.415	-3.626	0.075	2.37E-05	1.000	cg16026114	-2.060	0.449	0.577	4.35E-08	0.016
cg11611950	-1.554	-5.546	0.021	6.11E-05	1.000	cg07573057	-2.616	0.767	0.630	4.86E-08	0.016
cg07707031	-1.344	-3.590	0.077	0.000114	1.000	cg26282047	-2.039	0.805	0.636	1.28E-06	0.230
cg10017888	1.393	-4.529	0.042	0.000127	1.000	cg00235934	-1.450	0.553	0.595	1.55E-06	0.230
cg22204915	-1.620	0.822	0.639	0.000164	1.000	cg14789214	-1.605	0.632	0.608	1.74E-06	0.230
cg02652260	-1.216	-3.880	0.064	0.000179	1.000	cg08647039	-1.812	0.619	0.606	2.83E-06	0.258
cg05856122	-1.569	-3.266	0.094	0.00024	1.000	cg01457986	-1.893	0.793	0.634	2.95E-06	0.258
ch.2.54406426R	-1.151	-2.413	0.158	0.000255	1.000	cg00102439	-1.593	0.578	0.599	3.41E-06	0.258
cg08045063	-0.998	-4.236	0.050	0.000267	1.000	cg09964873	-1.594	0.679	0.616	3.52E-06	0.258
cg18881962	-1.951	-3.468	0.083	0.000285	1.000	cg17975896	-1.589	0.645	0.610	4.70E-06	0.287

logFC: log-fold change. Adj.p: FDR-corrected p-value

Table S6. **Effect of *MTHFR* variation on methylation-relevant metabolite levels in PBMC and muscle samples. Related to Figure 6 and Figure 7.**

**Metabolite**	**PBMC**				**Muscle**			
	Baseline		Day 30		Baseline		Day 30	
	MTHFR+	MTHFR-	MTHFR+	MTHFR-	MTHFR+	MTHFR-	MTHFR+	MTHFR-
**MeNAM**	0.107	0.133	1.394	0.940	2.139	1.834	5.477	4.266
**Adenosine**	4.960	5.085	4.961	4.266	0.875	0.802	0.903	0.846
**Homocysteine**	1.024	0.980	1.211	1.141	1.706	1.747	1.858	2.158
**NAM**	115.83	116.05	123.06	109.82	368.88	359.47	358.45	368.80
**Nicotinamide N-oxide**	NA	NA	NA	NA	0.007	0.010	0.058	0.056
**SAH**	1.857	2.320	2.429	1.909	1.938	1.995	1.839	1.938
**SAM**	0.583	0.562	0.672	0.403	7.075	8.084	5.741	7.089

Metabolomics data are taken from Brakedal et al[1] and reanalyzed according to *MTHFR* status in this study. The unit of measurement is pmol/10^6 cells for all PBMC values, and nmol/g for all muscle values. The interaction effect between *MTHFR* genotype and time (before and after NR treatment) was assessed by using a two-way repeated measures ANOVA, and there were no statistically significant effects (see main paper, Figure 6 and Figure 7). MTHFR+: individuals heterozygous for either the C677T or A1298C mutation; MTHFR-: individuals with wild type *MTHFR* status; NA: not available.

Table S7. **Subject characteristics. Related to STAR Methods – Experimental model and subject details – Cohorts**

Participant	Age	Sex	Group
02	65	Female	NR
05	66	Female	NR
06	64	Male	NR
10	72	Male	NR
11	69	Female	NR
13	69	Male	NR
15	44	Male	NR
19	60	Male	NR
20	50	Female	NR
22	63	Male	NR
24	73	Male	NR
28	65	Male	NR
30	64	Male	NR
31	73	Male	NR
01	66	Male	Placebo
03	66	Male	Placebo
04	65	Male	Placebo
07	55	Male	Placebo
09	68	Female	Placebo
12	65	Male	Placebo
14	61	Male	Placebo
16	63	Male	Placebo
18	60	Male	Placebo
21	75	Male	Placebo
23	69	Male	Placebo
25	74	Male	Placebo
26	73	Male	Placebo
27	29	Male	Placebo
29	66	Male	Placebo

References

1. Brakedal, B., Dölle, C., Riemer, F., Ma, Y., Nido, G.S., Skeie, G.O., Craven, A.R., Schwarzlmüller, T., Brekke, N., Diab, J., et al. (2022). The NADPARK study: A randomized phase I trial of nicotinamide riboside supplementation in Parkinson's disease. Cell Metabolism *34*, 396-407.e6. 10.1016/j.cmet.2022.02.001.
